# Digenic Origin of Difference of Sex Development in a Patient Harbouring DHX37 and MAMLD1 Variants

**DOI:** 10.1155/2024/4896940

**Published:** 2024-06-12

**Authors:** Katia Margiotti, Francesco Libotte, Marco Fabiani, Alvaro Mesoraca, Claudio Giorlandino

**Affiliations:** ^1^ALTAMEDICA, Human Genetics Lab, Viale Liegi 45, Rome 00198, Italy; ^2^ALTAMEDICA, Fetal-Maternal Medical Centre, Department of Prenatal Diagnosis, Viale Liegi 45, Rome 00198, Italy

## Abstract

**Background:**

The diagnostic process for identifying variations in sex development (DSD) remains challenging due to the limited availability of evidence pertaining to the association between phenotype and genotype. DSD incidence is reported as 2 in 10,000 births, and the etiology has been attributed to genetic causes. *Case Presentation*. The present study investigated genetic causes implicated in a case of a 15-year-old 46, XY patient, raised as a girl. Genetic analysis by clinical exome sequencing (CES) showed a digenic inheritance due to two known pathogenic mutations in the DHX37 gene and the MAMLD1 gene, while we excluded variants with pathogenic significance in 209 DSD-related genes.

**Conclusions:**

Based on our literature review, this is the first case with the combined presence of pathogenic mutations in the MAMLD1 gene and DHX37 gene in a patient with gonadal dysgenesis.

## 1. Introduction

In the year 2006, a novel terminology, namely, “disorders of sex development” (DSDs), was introduced. This term encompasses a group of congenital conditions characterized by atypical development of chromosomes, gonads, or anatomical sex [1]. The genetic cause of DSD still cannot be determined in about half of the cases. DSD is characterized by a wide clinical severity spectrum, ranging from genital ambiguity to moderate hypospadias or unilateral cryptorchidism to phenotypes that are so attenuated that they can go unnoticed. Complex genetic networks and hormonal signalling govern the development of the gonads. Comprehensive genetic testing is widely acknowledged as an essential element in the assessment of individuals with DSDs, owing to the intricate nature of gonad production and differentiation. Disorders of sex development comprise a wide range of clinical presentations that can be identified at various stages of life, spanning from the newborn period to late adulthood. However, a main common clinical characteristic observed in nearly all cases is infertility. The estimated incidence of severe 46, XY and 46, XX DSD with uncertain sex is 2.2 per 10,000 births [2, 3]. The observed syndrome exhibits a spectrum of manifestations, including variations in genital development such as ambiguous genitalia, moderate hypospadias, or unilateral cryptorchidism. Consequently, categorizing patients with similar or nearly identical phenotypes becomes challenging due to the presence of diverse etiologies and genetic mechanisms underlying this condition [4]. Numerous fundamental factors have been identified as possible causes, including mutations in genes encoding proteins involved in sex determination and development as well as genital development [5]. Nevertheless, the assessment of genotype-phenotype relationships remains challenging because of the considerable variability in both phenotypic and genotypic characteristics observed among people. Next-generation sequencing (NGS) technology has led to the identification of several novel DSD-causing genes and an improved understanding of the genetic basis and therapeutic management of the disease [6, 7]. Much is still unclear about the transmission of this pathology; mono- and oligogenic models are hypothesized [8]. The purpose of this study was to identify the genetic causes of a disorder of sex development in a case of an Albanian patient with 46, XY, and female external genitalia. We conducted NGS analysis and described known variants in the DEAH-box RNA helicase, DHX37 gene, and in mastermind-like domain-containing protein 1, MAMLD1 gene, revealing potential digenic inheritance origins in this DSD patient.

## 2. Case Presentation

A blood sample of a 15-year-old female patient raised as a female, arrived in our laboratory from Albania, and the major complaint was primary amenorrhea. The phenotype was described as follows: the presence of normal external genitalia, labia majora and labia minora, clitoris, and mons pubis, and the presence of normal hairs on the pubis indicating a spontaneous pubarche and no thelarche was reported. Pelvic ultrasounds reveal the absence of uterus. No hormones and/or gonadal tissue information were obtained. The parents were both healthy and nonconsanguineous. Following the medical indications, karyotyping was performed. Written consent was obtained from the carers or guardians on behalf of the participating minors. Genomic DNA was extracted from peripheral blood. The chromosome karyotyping analysis of the blood sample was performed using GTG-banding techniques on the PHA-stimulated blood lymphocytes. Cytogenetic examination and quantitative fluorescent polymerase chain reaction (QF-PCR, data not shown) indicated a male sex. To confirm the presence and function of the SRY gene, a FISH microdeletion PCR and a SRY sequencing were performed. The array comparative genomic hybridization (aCGH) analysis was performed using the 44K platform (Agilent Technologies) on DNA from blood to characterize the presence of DNA deletions or duplications. The results of the aCGH were negative, indicating no gain or loss of genetic material in the patient. After the aCGH, clinical exome sequencing (CES) by next-generation sequencing (NGS) (Illumina NextSeq500) was performed.

**Figure 1:**
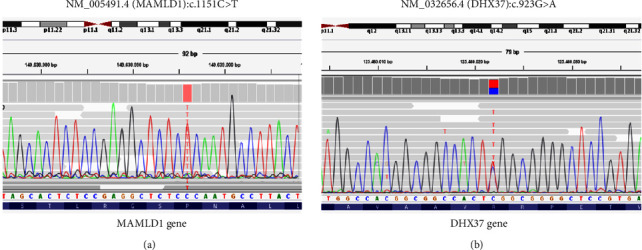
Sanger sequencing of the proband showing the two distinct identified variants. (a) Sanger sequencing of the sample, revealing the presence of the hemizygosis mutation c.1151C > T at the specific point indicated by the red rectangle. (b) The graph represents the Sanger sequencing of the heterozygous c.923G > A indicated by the red and blue rectangles. The identified variants are visualized by Integrative Genomics Viewer (IGV) software. Ref Seq (reference sequencing) used for variants annotation are NM_032656.4 for DHX37 and NM_005491.4 for MAMLD1 genes.

CES was carried out using the TruSight One Sequencing Panel (Illumina). The panel covers 4813 disease-associated genes. By using the bioinformatic tool eVai software (https://www.engenome.com) on the CES results, we were able to prioritize more than one variant in a DSD-related gene. The patient had a known variant in the DHX37 gene in combination with another known variant in the MAMLD1 gene (Figure 1).

By using DisGeNET (https://www.disgenet.org/home/), a discovery platform containing one of the largest publicly available collections of genes and variants associated with human diseases, we were able to select 209 genes related to several DSD phenotypic terms (gonadal dysgenesis, sex chromosome aberrations, disorders of sex development, congenital absence of ovary, congenital absence of germinal epithelium of testes, and gonadal dysgenesis 46 XY) [9]. No pathologic, likely pathologic, or uncertain variants were present in these genes associated with DSD (Supplementary Table 1). DEAH-box RNA helicase (DHX37) is an autosomal gene on chromosome 12q24.31. It is made up of 27 exons that code for a putative 1157-amino acid RNA helicase from the DExD/H-box helicases family. The pathogenic variant identified in the DHX37 gene is a missense substitution of arginine with glutamine at position 308 of the coding region (p.R308Q and NM_032656.4). The p.R308Q pathogenic variation, which occurs repeatedly, has been demonstrated to reduce the interaction between DNA/RNA at the binding site Ia of the RecA1 domain, consequently diminishing the helicase's ATPase activity [10]. Mastermind-like domain-containing 1 gene (MAMLD1), also known as chromosome X open reading frame 6 (CXorf6) or F18 (OMIM# 300120), was first identified in two patients with myotubular myopathy and male hypogenitalism, who were discovered to possess a deletion on chromosome Xq28 [11, 12]. The hemizygous variant identified in the MAMLD1 gene is a missense substitution of proline with leucine at position 384 of the coding region (p.P384L and NM_005491.4), and it has been shown that the 3D structure prediction of the mutated protein is altered and functional studies confirmed the significantly reduced transactivation function of the p.P384L protein [13]. Genetic analysis of the healthy parents was not conducted since they were not available for genetic testing.

## 3. Discussion

DSD is a significant pediatric concern, occurring in an estimated 1.7% of live deliveries [14]. In a hospital setting, a clinical genetic diagnosis is only made in 13% of the DSD patients, according to a previous study [15]. However, CES is now rapidly becoming a standard assay for the molecular diagnosis of rare disorders and has been used successfully on cohorts of DSD patients [7, 16–18]. Currently, a considerable number of genes have been linked to 46, XY DSD, such as, SRY, TSPYL1, WNT4, WT1, WWOX, ZFPM2, AKR1C2, AKR1C4, AMH, CYB5A, CYP11A1, CYP17A1, HSD17B3, HSD3B2, LHCGR, POR, and AR [7]. CES has provided evidence indicating that some patients with disorders of sex development (DSD) had more than one pathogenic or potentially pathogenic variation in genes that are known to be linked with sex development. Multiple variants of unknown significance in known DSD genes were also discovered in oligogenic combinations [8, 19]. Here, we describe a 46, XY patient with female external genitalia, an absent uterus, and no thelarche. The patient harbored a photogenic variant in the DHX37 gene (p.R308Q) together with a hemizygous variant in the MAMLD1 gene (p.P384L). In 2019, heterozygous missense variants in DHX37 (OMIM ^*∗*^617362) were described in individuals with 46, XY gonadal dysgenesis or testicular regression syndrome, becoming a new gene associated with 46, XY DSD [20]. This gene has been linked to approximately 16% of all cases of DSD [21]. So far, 16 DHX37 variants have been identified in 43 individuals with distinct 46, XY DSD phenotypes [21]. A single individual was documented as being homozygous, suggesting the presence of an autosomal dominant mode of inheritance. Prior to these findings, it was shown that homozygous or compound heterozygous variants in the DHX37 gene were associated with neurodevelopmental disorders characterized by brain abnormalities, as well as potential vertebral or cardiac problems [22]. Phenotypically, patients carrying the recurrent pathogenic variant, p.R308Q, varied from female to male phenotype [20]. For example, Mcelreavey et al. [20] identified five children carrying the p.R308Q pathogenic variant with a phenotype that varied from female with primary amenorrhea to male with micropenis and bilateral cryptorchidism, rendering genotype/phenotype correlation very difficult and confirming that variants in DHX37 are not solely responsible for the clinical phenotype. As a potential contribution to the phenotype of the discussed case, we also found a hemizygous variant c.1151C > T mutation (p.P384L) in exon 2 of the MAMLD1 gene. Variants in the MAMLD1 gene have been associated with a broad range of DSD phenotypes, mainly hypospadias [23]. Other phenotypes include cryptorchidism, micropenis, complete female external genitalia, and primary amenorrhea [23–26]. Several studies have shown in DSD patients (both 46, XY and 46, XX) MAMLD1 variants in combination with other mutations in DSD-related genes [11]. Moreover, it has been shown that some MAMLD1 variants are carried by unaffected individuals, indicating that MAMLD1 variants lead to DSD in combination with other genetic abnormalities [27, 28].

While MAMLD1 variants are found in the general population without any apparent phenotypic consequences, their presence in DSD patients with diverse manifestations has raised questions about their direct causative role. This observation suggests a more nuanced genetic landscape, where MAMLD1 variants alone may not fully account for the observed DSD phenotypes. Both MAMLD1 and DHX37 play crucial roles in DSD phenotypes, yet direct interaction or interplay between these two genes specifically in the context of DSD has not been extensively documented. However, the data suggest that both genes are part of a broader network of genetic interactions affecting gonadal development. In particular, MAMLD1 is expressed in the testes during critical phases of embryonic development, suggesting its involvement in testicular organogenesis and androgen synthesis, which are crucial hormones for the development of primary and secondary male sexual characteristics [29]. The primary function of MAMLD1 appears to be linked to the regulation of key genes involved in the biosynthesis of testosterone, such as CYP17A1, an essential enzyme in steroid production [30]. Given these roles, the speculative interaction between MAMLD1 and DHX37 in DSD could lie in the broader context of cellular and developmental processes required for proper gonadal and genital development. For example, any dysregulation in ribosome biogenesis (potentially caused by dysfunctional DHX37) could affect the cellular environment in which MAMLD1 operates, thereby influencing its regulatory effects on target genes essential for sex development. This might not represent a direct gene-gene interaction but rather a cascade effect where the normal functioning of each gene is pivotal for the pathway that leads to the correct sexual development and differentiation. Such an interaction suggests a complex network where the pathology observed in DSD might result from compounded minor disruptions in various processes, each contributed by different genes such as MAMLD1 and DHX37.

Interestingly, supporting the concept of digenic inheritance in DSD phenotype, de Oliveira et al. [21] described a case involving a 46, XY patient with female external genitalia and nonpalpable gonads. This patient exhibited pathogenic variants in both the NR5A1 and DHX37 genes. This finding was corroborated by the fact that the parents exhibited identical mutations in both genes (the mother carried the NR5A1 variant and the father carried the DHX37 variant) but did not exhibit any symptoms [21].

Finally, we could exclude the presence of potentially deleterious or candidate variants in the most relevant DSD-related gene (Supplementary Table 1).

## 4. Conclusion

To our best knowledge, the MAMLD1 gene and DHX37 gene pathogenic variants have never been identified together in a patient with 46, XY gonadal dysgenesis, raising the possibility of a digenic inherited origin in this case study. In conclusion, the complete female types of external genitalia seen in this patient might be associated with DHX37 and MAMLD1 genes pathogenic variant combinations even though we cannot exclude other factors that might contribute to the phenotypic outcome. Further evidence of the DHX37-MAMLD1 digenic origin of 46, XY DSD phenotypes has yet to be identified to better define the synergistic effect of these two genes.

## Data Availability

The data used to support the findings of this study are included within the article.

## Supplementary Materials

Supplementary Materials DisGeNET analysis revealed 209 genes related to several DSD phenotypic terms (gonadal dysgenesis, sex chromosome aberrations, disorders of sex development, congenital absence of ovary, and congenital absence of germinal epithelium of testes, gonadal dysgenesis 46 XY) [9].
