# Supplementary material for: Digenic Origin of Difference of Sex Development in a Patient Harbouring DHX37 and MAMLD1 Variants
Source: Case Rep Pediatr. 2024 Jun 12;2024:4896940. doi: 10.1155/2024/4896940 (PMC11221946; doi:10.1155/2024/4896940)
Supplement: Supplementary Materials — DisGeNET analysis revealed 209 genes related to several DSD phenotypic terms (gonadal dysgenesis, sex chromosome aberrations, disorders of sex development, congenital absence of ovary, and congenital absence of germinal epithelium of testes, gonadal dysgenesis 46 XY) [9]. [file 4896940.f1.docx]

**Supplementary Table 1**

DisGeNET analysis revealed 209 genes related to several DSD phenotypic terms (Gonadal Dysgenesis ,Sex Chromosome Aberrations, Disorders of Sex Development, Congenital absence of ovary, and Congenital absence of germinal epithelium of testes, Gonadal Dysgenesis 46 XY) (Pinero et al. 2021).

| Disease | Gene | Gene_id |
| --- | --- | --- |
| Gonadal Dysgenesis | NR5A1 | 2516 |
| Gonadal Dysgenesis | NUP107 | 57122 |
| Gonadal Dysgenesis | FMR1 | 2332 |
| Gonadal Dysgenesis | SRY | 6736 |
| Gonadal Dysgenesis | WT1 | 7490 |
| Gonadal Dysgenesis | MAP3K1 | 4214 |
| Gonadal Dysgenesis | SOX9 | 6662 |
| Gonadal Dysgenesis | NR0B1 | 190 |
| Gonadal Dysgenesis | ZFPM2 | 23414 |
| Gonadal Dysgenesis | SPIDR | 23514 |
| Gonadal Dysgenesis | RXYLT1 | 10329 |
| Gonadal Dysgenesis | TOE1 | 114034 |
| Gonadal Dysgenesis | MPLKIP | 136647 |
| Gonadal Dysgenesis | ERCC2 | 2068 |
| Gonadal Dysgenesis | ERCC3 | 2071 |
| Gonadal Dysgenesis | FGFR3 | 2261 |
| Gonadal Dysgenesis | FSHR | 2492 |
| Gonadal Dysgenesis | GATA4 | 2626 |
| Gonadal Dysgenesis | GTF2E2 | 2961 |
| Gonadal Dysgenesis | PSMC3IP | 29893 |
| Gonadal Dysgenesis | HSD17B4 | 3295 |
| Gonadal Dysgenesis | KIT | 3815 |
| Gonadal Dysgenesis | GTF2H5 | 404672 |
| Gonadal Dysgenesis | DHH | 50846 |
| Gonadal Dysgenesis | WWOX | 51741 |
| Gonadal Dysgenesis | TWNK | 56652 |
| Gonadal Dysgenesis | MRPS22 | 56945 |
| Gonadal Dysgenesis | DMRT3 | 58524 |
| Gonadal Dysgenesis | STK11 | 6794 |
| Gonadal Dysgenesis | VAMP7 | 6845 |
| Gonadal Dysgenesis | RNF113A | 7737 |
| Gonadal Dysgenesis | BCL10 | 8915 |
| Gonadal Dysgenesis | BMP15 | 9210 |
| Gonadal Dysgenesis | DMRT1 | 1761 |
| Gonadal Dysgenesis | AMH | 268 |
| Gonadal Dysgenesis | MAMLD1 | 10046 |
| Gonadal Dysgenesis | PAICS | 10606 |
| Gonadal Dysgenesis | DMRT2 | 10655 |
| Gonadal Dysgenesis | CYP21A2 | 1589 |
| Gonadal Dysgenesis | FANCA | 2175 |
| Gonadal Dysgenesis | FGF9 | 2254 |
| Gonadal Dysgenesis | GART | 2618 |
| Gonadal Dysgenesis | SOX8 | 30812 |
| Gonadal Dysgenesis | HLA-A | 3105 |
| Gonadal Dysgenesis | AR | 367 |
| Gonadal Dysgenesis | PPP2R3C | 55012 |
| Gonadal Dysgenesis | LHX9 | 56956 |
| Gonadal Dysgenesis | DHX37 | 57647 |
| Gonadal Dysgenesis | SHBG | 6462 |
| Gonadal Dysgenesis | SRD5A2 | 6716 |
| Gonadal Dysgenesis | SSRP1 | 6749 |
| Gonadal Dysgenesis | PPIG | 9360 |
| Gonadal Dysgenesis | CD38 | 952 |
| Disorders of Sex Development | HSD17B3 | 3293 |
| Disorders of Sex Development | LHCGR | 3973 |
| Disorders of Sex Development | AKR1C1 | 1645 |
| Disorders of Sex Development | AKR1C2 | 1646 |
| Disorders of Sex Development | DHCR7 | 1717 |
| Disorders of Sex Development | PPP1R12A | 4659 |
| Disorders of Sex Development | AKR1C3 | 8644 |
| Disorders of Sex Development | NR5A1 | 2516 |
| Disorders of Sex Development | SRY | 6736 |
| Disorders of Sex Development | SOX9 | 6662 |
| Disorders of Sex Development | AR | 367 |
| Disorders of Sex Development | STAR | 6770 |
| Disorders of Sex Development | CYP2B6 | 1555 |
| Disorders of Sex Development | AMH | 268 |
| Disorders of Sex Development | RSPO1 | 284654 |
| Disorders of Sex Development | SOX10 | 6663 |
| Disorders of Sex Development | SRD5A2 | 6716 |
| Disorders of Sex Development | TSPY1 | 7258 |
| Disorders of Sex Development | TSPY10 | 1E+08 |
| Disorders of Sex Development | MAMLD1 | 10046 |
| Disorders of Sex Development | GADD45G | 10912 |
| Disorders of Sex Development | CYP11A1 | 1583 |
| Disorders of Sex Development | CYP17A1 | 1586 |
| Disorders of Sex Development | CYP19A1 | 1588 |
| Disorders of Sex Development | DECR1 | 1666 |
| Disorders of Sex Development | DMRT1 | 1761 |
| Disorders of Sex Development | FGF9 | 2254 |
| Disorders of Sex Development | FOXF2 | 2295 |
| Disorders of Sex Development | QPCT | 25797 |
| Disorders of Sex Development | GATA4 | 2626 |
| Disorders of Sex Development | KLK3 | 354 |
| Disorders of Sex Development | MAP3K4 | 4216 |
| Disorders of Sex Development | WWOX | 51741 |
| Disorders of Sex Development | HHAT | 55733 |
| Disorders of Sex Development | TSPYL1 | 7259 |
| Disorders of Sex Development | TSPY3 | 728137 |
| Disorders of Sex Development | WNT5A | 7474 |
| Disorders of Sex Development | CBX2 | 84733 |
| Disorders of Sex Development | PPIG | 9360 |
| Gonadal Dysgenesis, 46,XY | SRY | 6736 |
| Gonadal Dysgenesis, 46,XY | NR5A1 | 2516 |
| Gonadal Dysgenesis, 46,XY | SOX9 | 6662 |
| Gonadal Dysgenesis, 46,XY | DMRT1 | 1761 |
| Gonadal Dysgenesis, 46,XY | ZFPM2 | 23414 |
| Gonadal Dysgenesis, 46,XY | MAP3K1 | 4214 |
| Gonadal Dysgenesis, 46,XY | DHH | 50846 |
| Gonadal Dysgenesis, 46,XY | WT1 | 7490 |
| Gonadal Dysgenesis, 46,XY | CBX2 | 84733 |
| Gonadal Dysgenesis, 46,XY | DHX37 | 57647 |
| Gonadal Dysgenesis, 46,XY | TSPY10 | 1E+08 |
| Gonadal Dysgenesis, 46,XY | CTNNB1 | 1499 |
| Gonadal Dysgenesis, 46,XY | NR0B1 | 190 |
| Gonadal Dysgenesis, 46,XY | F8 | 2157 |
| Gonadal Dysgenesis, 46,XY | FGF9 | 2254 |
| Gonadal Dysgenesis, 46,XY | FLNB | 2317 |
| Gonadal Dysgenesis, 46,XY | GATA4 | 2626 |
| Gonadal Dysgenesis, 46,XY | AMH | 268 |
| Gonadal Dysgenesis, 46,XY | SGSM3 | 27352 |
| Gonadal Dysgenesis, 46,XY | PBX1 | 5087 |
| Gonadal Dysgenesis, 46,XY | PPP2R3C | 55012 |
| Gonadal Dysgenesis, 46,XY | RAC1 | 5879 |
| Gonadal Dysgenesis, 46,XY | SOX3 | 6658 |
| Gonadal Dysgenesis, 46,XY | TSPY1 | 7258 |
| Gonadal Dysgenesis, 46,XY | TSPY3 | 728137 |
| Gonadal Dysgenesis, 46,XY | ZFY | 7544 |
| Gonadal Dysgenesis, 46,XY | DDX3Y | 8653 |
| Gonadal Dysgenesis, 46,XY | FAM189A2 | 9413 |
| Gonadal Dysgenesis, 46,XY | STARD8 | 9754 |
| Congenital absence of ovary | DIPK1A | 388650 |
| Congenital absence of ovary | PMM2 | 5373 |
| Congenital absence of ovary | PTPN11 | 5781 |
| Congenital absence of ovary | RPL5 | 6125 |
| Congenital absence of ovary | TP63 | 8626 |
| Congenital absence of germinal epithelium of testes | DMC1 | 11144 |
| Congenital absence of germinal epithelium of testes | NUPR1 | 26471 |
| Congenital absence of germinal epithelium of testes | DAZ1 | 1617 |
| Congenital absence of germinal epithelium of testes | BRD2 | 6046 |
| Congenital absence of germinal epithelium of testes | DDX3Y | 8653 |
| Congenital absence of germinal epithelium of testes | AZF1 | 560 |
| Congenital absence of germinal epithelium of testes | NR0B1 | 190 |
| Congenital absence of germinal epithelium of testes | DAZ2 | 57055 |
| Congenital absence of germinal epithelium of testes | TSPY10 | 1E+08 |
| Congenital absence of germinal epithelium of testes | CDK2 | 1017 |
| Congenital absence of germinal epithelium of testes | PLK4 | 10733 |
| Congenital absence of germinal epithelium of testes | CFTR | 1080 |
| Congenital absence of germinal epithelium of testes | CYP2R1 | 120227 |
| Congenital absence of germinal epithelium of testes | SPATA17 | 128153 |
| Congenital absence of germinal epithelium of testes | CYP11A1 | 1583 |
| Congenital absence of germinal epithelium of testes | CYP17A1 | 1586 |
| Congenital absence of germinal epithelium of testes | CYP19A1 | 1588 |
| Congenital absence of germinal epithelium of testes | HSFY2 | 159119 |
| Congenital absence of germinal epithelium of testes | CDY2B | 203611 |
| Congenital absence of germinal epithelium of testes | ETV5 | 2119 |
| Congenital absence of germinal epithelium of testes | FANCA | 2175 |
| Congenital absence of germinal epithelium of testes | FGF5 | 2250 |
| Congenital absence of germinal epithelium of testes | FGF9 | 2254 |
| Congenital absence of germinal epithelium of testes | HSPA4L | 22824 |
| Congenital absence of germinal epithelium of testes | SIN3A | 25942 |
| Congenital absence of germinal epithelium of testes | GAPDH | 2597 |
| Congenital absence of germinal epithelium of testes | AMH | 268 |
| Congenital absence of germinal epithelium of testes | GJA1 | 2697 |
| Congenital absence of germinal epithelium of testes | PABPC1 | 26986 |
| Congenital absence of germinal epithelium of testes | DKKL1 | 27120 |
| Congenital absence of germinal epithelium of testes | GNRHR | 2798 |
| Congenital absence of germinal epithelium of testes | APC | 324 |
| Congenital absence of germinal epithelium of testes | HSD3B2 | 3284 |
| Congenital absence of germinal epithelium of testes | HSPA2 | 3306 |
| Congenital absence of germinal epithelium of testes | HSPB1 | 3315 |
| Congenital absence of germinal epithelium of testes | HSPB2 | 3316 |
| Congenital absence of germinal epithelium of testes | IGF1 | 3479 |
| Congenital absence of germinal epithelium of testes | IL1RN | 3557 |
| Congenital absence of germinal epithelium of testes | FASLG | 356 |
| Congenital absence of germinal epithelium of testes | ING2 | 3622 |
| Congenital absence of germinal epithelium of testes | INHBB | 3625 |
| Congenital absence of germinal epithelium of testes | INSL3 | 3640 |
| Congenital absence of germinal epithelium of testes | LRP6 | 4040 |
| Congenital absence of germinal epithelium of testes | MIR133B | 442890 |
| Congenital absence of germinal epithelium of testes | CLDN11 | 5010 |
| Congenital absence of germinal epithelium of testes | PABPC3 | 5042 |
| Congenital absence of germinal epithelium of testes | PGAM1 | 5223 |
| Congenital absence of germinal epithelium of testes | PIK3CA | 5290 |
| Congenital absence of germinal epithelium of testes | PIK3CB | 5291 |
| Congenital absence of germinal epithelium of testes | PIK3CD | 5293 |
| Congenital absence of germinal epithelium of testes | PIK3CG | 5294 |
| Congenital absence of germinal epithelium of testes | DDX4 | 54514 |
| Congenital absence of germinal epithelium of testes | LRRC8A | 56262 |
| Congenital absence of germinal epithelium of testes | PRPS2 | 5634 |
| Congenital absence of germinal epithelium of testes | PRS | 5640 |
| Congenital absence of germinal epithelium of testes | KNL1 | 57082 |
| Congenital absence of germinal epithelium of testes | ADGRG6 | 57211 |
| Congenital absence of germinal epithelium of testes | MIR202 | 574448 |
| Congenital absence of germinal epithelium of testes | FANCM | 57697 |
| Congenital absence of germinal epithelium of testes | CCND1 | 595 |
| Congenital absence of germinal epithelium of testes | BCL2 | 596 |
| Congenital absence of germinal epithelium of testes | RAD21L1 | 642636 |
| Congenital absence of germinal epithelium of testes | SNRNP70 | 6625 |
| Congenital absence of germinal epithelium of testes | TSPY1 | 7258 |
| Congenital absence of germinal epithelium of testes | TSPY3 | 728137 |
| Congenital absence of germinal epithelium of testes | DDR1 | 780 |
| Congenital absence of germinal epithelium of testes | NPHS2 | 7827 |
| Congenital absence of germinal epithelium of testes | ESX1 | 80712 |
| Congenital absence of germinal epithelium of testes | USP9Y | 8287 |
| Congenital absence of germinal epithelium of testes | CASP3 | 836 |
| Congenital absence of germinal epithelium of testes | TEX101 | 83639 |
| Congenital absence of germinal epithelium of testes | SPATA9 | 83890 |
| Congenital absence of germinal epithelium of testes | SPATA16 | 83893 |
| Congenital absence of germinal epithelium of testes | C9orf24 | 84688 |
| Congenital absence of germinal epithelium of testes | RANBP3 | 8498 |
| Congenital absence of germinal epithelium of testes | HSFY1 | 86614 |
| Congenital absence of germinal epithelium of testes | CCNA1 | 8900 |
| Congenital absence of germinal epithelium of testes | HSPB3 | 8988 |
| Congenital absence of germinal epithelium of testes | BPY2 | 9083 |
| Congenital absence of germinal epithelium of testes | CDY1 | 9085 |
| Congenital absence of germinal epithelium of testes | CDY2A | 9426 |
| Congenital absence of germinal epithelium of testes | REC8 | 9985 |
